# Prevalence of genetic alterations in basal cell carcinoma patients resistant to Hedgehog pathway inhibitors: a systematic review

**DOI:** 10.1080/07853890.2025.2516701

**Published:** 2025-06-16

**Authors:** Suvijak Untaaveesup, Pornteera Srichana, Gynna Techataweewan, Chanamon Pongphaew, Wichapol Dendumrongsup, Ben Ponvilawan, Nichanant Nampipat, Chanin Limwongse

**Affiliations:** ^a^ Chao Khun Paiboon Hospital, Kanchanaburi, Thailand; ^b^ Detudom Crown Prince Hospital, Ubon Ratchathani, Thailand; ^c^ Faculty of Medicine, Burapha University Hospital, Burapha University, Chonburi, Thailand; ^d^ Princess Srisavangavadhana College of Medicine, Chulabhorn Royal Academy, Bangkok, Thailand; ^e^ Faculty of Medicine, Chulalongkorn University, Bangkok, Thailand; ^f^ Department of Internal Medicine, University of Missouri–Kansas City School of Medicine, Kansas City, MO, USA; ^g^ Department of Medicine, Medical Oncology Unit, Maha Vajiralongkorn Thanyaburi Hospital, Pathum Thani, Thailand; ^h^ Department of Medicine, Division of Medical Genetics, Faculty of Medicine Siriraj Hospital, Mahidol University, Bangkok, Thailand

**Keywords:** Basal cell carcinoma, genetic alteration, Hedgehog pathway inhibitor, prevalence, systematic review

## Abstract

**Introduction:**

Basal cell carcinoma (BCC) is a prevalent form of skin cancer that can be localized or metastatic. Current evidence supports the use of Hedgehog (Hh) pathway inhibitors for locally advanced or metastatic BCC with resistance due to genetic alterations in the Hh pathway. This systematic review evaluated the prevalence of genetic alterations in Hh pathway genes in BCC.

**Materials and methods:**

We conducted a comprehensive search across four databases: PubMed, EMBASE, SCOPUS and the Cochrane Library. We included articles reporting genetic alterations in patients with locally advanced or metastatic BCC resistant to Hh pathway inhibitors.

**Results:**

We included three prospective cohort studies encompassing 27 samples, all of which were resistant to vismodegib treatment. The most prevalent genetic mutations in the Hh pathway were in *PTCH1*, *SMO* and *TP53*, with a pooled prevalence of 44.44%.

**Conclusions:**

This systematic review highlights the prevalence of genetic alterations in the Hh pathway in BCC and offers insights into the mechanisms involved in treatment resistance. Understanding the high resistance rates of these genes may facilitate the development of more effective targeted therapies for BCC.

## Key messages

The recent literature found no study established the prevalence and genetic alterations which related systemic treatments in treating advanced basal cell carcinoma. This study found that *PTCH1*, *SMO* and *TP53* were the most prevalent genetic mutations in the Hedgehog pathway. As a result, our results may influence further therapeutic strategies, resulting in disruption of the resistance mechanisms.

## Introduction

Basal cell carcinoma (BCC) is the most prevalent form of skin cancer, particularly among White people, especially Australians, and generally has a favourable prognosis. The primary risk factor is ultraviolet (UV) exposure, and aging also increases susceptibility. Genetic alterations in the Hedgehog (Hh) pathway significantly contribute to BCC tumour growth, notably through gain-of-function mutations [1–4]. The *Patched1* (*PTCH1*), *tumor protein 53* (*TP53*), *smoothened* (*SMO*) and *suppressor of fused* (*SUFU*) genes are frequently mutated in both sporadic and hereditary BCC, including Gorlin syndrome [3,5,6].

The standard treatment for BCC is surgical excision, including Mohs surgery. Other modalities are laser therapy, radiation therapy for nonadvanced BCC, and targeted therapies for locally advanced or metastatic BCC [7]. Hedgehog pathway inhibitors, such as vismodegib and sonidegib, are oral medications approved for treating locally advanced or metastatic BCC. These inhibitors target *SMO*, thereby reducing tumor size by disrupting the release of glioma-associated oncogene homolog 1 (Gli1) [3,7]. However, resistance to these treatments can develop due to genetic alterations, primarily in the *PTCH1*, *SMO*, *GLI* and *TP53* genes [4,6]. To date, no comprehensive study has examined the prevalence and specific genetic alterations in the patients who resistant to HHIs related to BCC.

Thus, this systematic review aimed to investigate the prevalence of genetic alterations in the patients who are resistant to HHIs and their association with BCC.

## Materials and methods

### Study design and registration

This systematic review was conducted according to the Preferred Reporting Items for Systematic Reviews and Meta-Analyses (PRISMA) guidelines, as elaborated in Supplementary Data 1. The study protocol was registered with the International Platform of Registered Systematic Review and Meta-analysis Protocols (INPLASY; registration number INPLASY2023120106) [8].

### Data source and search strategy

Seven researchers (S.U., P.S., G.T., C.P., W.D., B.P. and N.N.) independently conducted searches for eligible articles across four databases: PUBMED, EMBASE, SCOPUS and the Cochrane Library. The focus was on observational studies involving patients with locally advanced or metastatic BCC resistant to Hh pathway inhibitors, specifically vismodegib or sonidegib. The search terms used were ‘basal cell carcinoma,’ ‘vismodegib’ and ‘sonidegib,’ covering the period from database inception to October 2023. Supplementary Data 2 is depicted the search strategy. Additionally, the reference lists of the included articles were thoroughly reviewed to ensure that no relevant studies were overlooked.

### Selection criteria

The inclusion criteria for eligible studies were as follows:

- Participants had either locally advanced or metastatic BCC that was inoperable.
- All subtypes of BCC, such as basosquamous carcinoma (BSC), nodular or morpheaform subtypes, were included.
- Analyses were conducted of genetic alterations in tissues following surgical treatment.
- Treatment involved the Hh pathway inhibitors vismodegib or sonidegib.
- Prospective or retrospective cohort designs were employed.

The exclusion criteria for studies were the following:

- Participants had operable or nonlocally advanced BCC.
- Participants with Gorlin syndrome were excluded.
- Participants were treated with Hh pathway inhibitors before surgical intervention.
- Genetic alterations were analysed from tissues prior to targeted treatment.
- Designs were not cohort studies.

The literature review involved two independent steps performed by the seven researchers. First, topics and abstracts were reviewed, followed by a comprehensive review of full-text articles. Any disagreements were resolved through discussion among the researchers.

### Data extraction and quality assessment

Data extraction was independently performed using a predefined form. The following information was collected: author, year of publication, number of participants, study design, sex, mean age, study duration, list of gene mutations, and specific drug refractory to treatment. The quality of the included cohort studies was independently assessed by two researchers (S.U., P.S.) using the Newcastle–Ottawa Scale [9]. Disagreements were resolved through discussion.

## Results

### Screening process and article selection

**Figure 1.**
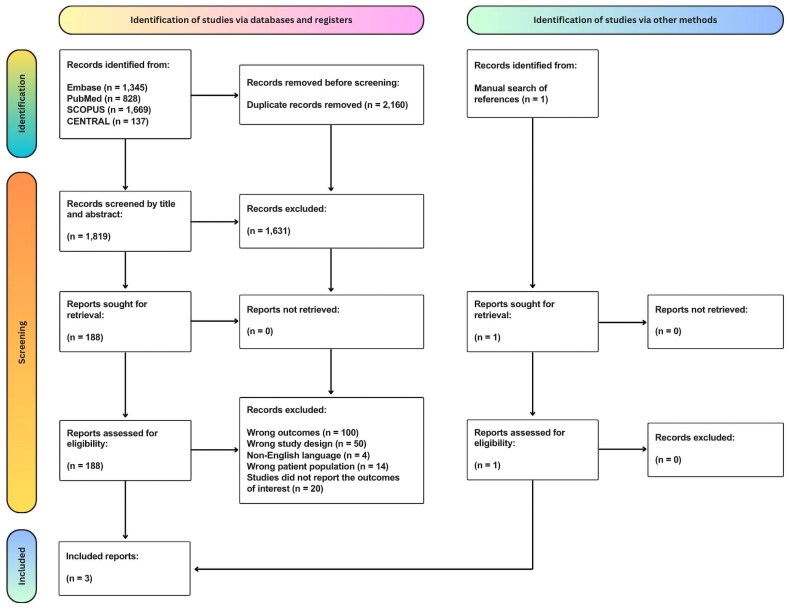
PRISMA flowchart for article selection and screening.

A total of 3980 potentially eligible articles were identified: 1345 from EMBASE, 828 from PubMed, 1669 from SCOPUS and one from other sources. We excluded 2160 duplicate articles, leaving 1819 articles screened by title and abstract. The full texts of 188 articles were subsequently reviewed, and most were excluded due to irrelevant outcomes, incorrect study designs, non-English languages, incorrect patient populations or a lack of relevant outcome data. Ultimately, three prospective cohort studies were included in the final systematic review [2,6,10]. The screening process is depicted in Figure 1.

### Baseline characteristics of included studies

This systematic review included three prospective cohort studies encompassing 27 samples, all of which exhibited resistance to vismodegib treatment. The baseline characteristics and quality assessments of the included studies are detailed in Table 1. Two studies involving 19 patients reported acquired resistance to vismodegib [6,10]. Of these, Bonilla et al. [6] investigated the genetic alterations in vismodegib-resistant BCC, incorporating samples from Atwood et al. [4] and Sharpe et al. [11]. Hence, we excluded Atwood et al. [4] and Sharpe et al. [11] from our systematic review.

**Table 1.** Baseline characteristics and quality assessment of included studies.

Study	Number of patients (samples)	Study design	Sex (male/female)	Mean age (years)	Subtype of BCC	Duration of study	List gene (*n*)	Refractory to treatment	Newcastle–Ottawa scale
*Acquired resistance*									
Bonilla et al. [6]	14 (20)	P	NA	NA	Nodular (2/14), NA (12/14)	NA	PTCH1 (9/14), SMO (6/14), SUFU (3/14), TP53 (8/14), MYCN (6/14)	Vismodegib	Selection: 4, comparability: 0, outcome: 2
Danial et al. [10]	5 (8)	P	8 (7/1)	57.8	NA	2011–2013	SMO (5/8)	Vismodegib	Selection: 3, comparability: 0, outcome: 2
*Intrinsic resistance*									
Yurchenko et al. [2]	(5)	P	5 (4/1)	NA	Basosquamous (4/5), morpheaform (1/5)	2011–2013	PTCH1 (3/5), SMO (1/5), TP53 (4/5), KMT2D (4/5), ATRX (1/5), ARID1A (1/5), KDM6A (1/5), TET2 (1/5), ASXL1 (1/5), SMAD4 (2/5), TGFBR1 (1/5), FAT1 (1/5), CTNNB1 (1/5), MYC (1/5), MYCN (2/5), ERBB4 (1/5), ERBB2 (1/5), MAP2K4 (1/5), PIK3CA (1/5), RAD50 (1/5), BIRC3 (1/5), KNSTRN (1/5), TSC2 (2/5), CIITA (1/5), PMS2 (1/5), CUX1 (1/5), RECQL4 (1/5), OPA1 (1/5), HLA-A (1/5), PARK2 (2/5), CRLF2 (1/5), CARD11 (1/5), RBM10 (1/5), MTOR (1/5), NSD1(1/5), FAM58A (1/5), DTX1 (1/5), NOTCH2 (1/5), EP400 (1/5), PTPRT (1/5), CTCF(1/5), NOTCH1 (1/5), BCL2L11 (1/5)	Vismodegib	Selection: 3, comparability: 0, outcome: 2

BCC: basal cell carcinoma; NA: not applicable; P: prospectively; R: retrospectively.

### Pooled prevalence of gene mutations in treatment-resistant BCC

The gene mutations within the Hh pathway from the included studies were analysed, as presented in Table 2. The most prevalent genetic mutations in vismodegib-resistant BCC were in *PTCH1*, *SMO* and *TP53*, each with a pooled prevalence of 44.44% [2,6,10]. Additionally, 29.63% of patients had *MYCN* mutations [2,6]. *KMT2D* mutations were noted with a pooled prevalence of 14.81% [2].

**Table 2.** Pooled prevalence of genetic alterations in basal cell carcinoma.

List gene mutations	Number of included articles	Number of samples/participants (*n*, %)
PTCH1	2	12, 44.44%
TP53	2	12, 44.44%
MYCN	2	8, 29.63%
SMO	3	12, 44.44%
SUFU	1	3, 11.11%
KMT2D	1	4, 14.81%
ATRX	1	1, 3.7%
ARID1A	1	1, 3.7%
KDM6A	1	1, 3.7%
TET2	1	1, 3.7%
ASXL1	1	1, 3.7%
SMAD4	1	2, 7.41%
TGFBR1	1	1, 3.7%
FAT1	1	1, 3.7%
CTNNB1	1	1, 3.7%
MYC	1	1, 3.7%
ERBB4	1	1, 3.7%
ERBB2	1	1, 3.7%
MAP2K4	1	1, 3.7%
PIK3CA	1	1, 3.7%
RAD50	1	1, 3.7%
BIRC3	1	1, 3.7%
KNSTRN	1	1, 3.7%
TSC2	1	2, 7.41%
CIITA	1	1, 3.7%
PMS2	1	1, 3.7%
CUX1	1	1, 3.7%
RECQL4	1	1, 3.7%
OPA1	1	1, 3.7%
HLA-A	1	1, 3.7%
PARK2	1	2, 7.41%
CRLF2	1	1, 3.7%
CARD11	1	1, 3.7%
RBM10	1	1, 3.7%
MTOR	1	1, 3.7%
NSD1	1	1, 3.7%
FAM58A	1	1, 3.7%
DTX1	1	1, 3.7%
NOTCH2	1	1, 3.7%
EP400	1	1, 3.7%
PTPRT	1	1, 3.7%
CTCF	1	1, 3.7%
NOTCH1	1	1, 3.7%
BCL2L11	1	1, 3.7%

## Discussion

This systematic review identified genetic alterations in Hh pathway genes such as *PTCH1*, *TP53*, *MYCN*, *SMO* and *SUFU* that confer resistance to Hh pathway inhibitors, notably vismodegib. These findings align with data from a cohort study indicating that *SMO* mutations are most prevalent in sporadic BCC tissues resistant to vismodegib [11]. This resistance could be attributed to the critical role of the Hh pathway in BCC pathogenesis despite its low affinity for *SMO* transducers and limited tumor development [4].

The incidence of BCC was likewise mostly depicted in Australia with an age-standardized rate of 37 per 100,000 population. This can be explained by intense UV radiation as a major precipitating factor of BCC in Australia due to the adjacency of the equator [12,13]. Additionally, the increasing trend of BCC was noticeably observed in high UV radiation areas [14]. UV exposure, which depends on the closeness of the equator, induces deoxyribonucleic acid (DNA) damage and *p53* gene alteration, also formulating BCC [12,13]. Furthermore, the light-skinned colour in Australians, mostly Caucasians, is also an incremental risk of BCC [15].

Previous studies have elucidated several mechanisms underlying resistance to Hh pathway inhibitors. First, atypical protein kinase C iota/lambda (aPKC) loss of function can inhibit Hh signalling [16]. Second, *Gli2* (a transcription factor) and cyclin D1 (Ccnd1) are amplified within the Hh pathway [17]. Third, the phosphoinositide 3-kinase (PI3K) pathway activates G protein-coupled receptor (GPCR)-like proteins [18]. Additionally, resistance can be intrinsic or acquired. Intrinsic resistance (resistance occurring at the initiation of drug therapy) was observed in a study of 148 locally advanced BCC patients who were treated with vismodegib 150 mg/day and monitored for at least 1 year. These patients exhibited high genomic instability, characterized by increases in the fraction of the genome altered, somatic copy number alterations and aneuploidy, compared to naive BCC patients through an unknown mechanism. Conversely, acquired resistance is defined as a tumor failing to respond to treatment after a period, resulting in relapse [2]. For example, one BCC patient treated with vismodegib 150 mg/day relapsed at 20 weeks, with pre- and posttreatment genetic analyses revealing *SMO* mutations after – but not before – treatment [19].

Genetic alterations in the Hh pathway that cause resistance to inhibitors have also been identified in other cancers, including medulloblastoma. In these studies, *SMO* mutations were noted after administering Hh pathway inhibitors. The resistance mechanism may involve GPCR-like proteins enhanced by the PI3K pathway, leading to tumor relapse [18,20].

Our systematic review excluded Gorlin syndrome, a hereditary cancer syndrome predominantly involving *PTCH1* mutations, to avoid skewing the results for non-Gorlin BCC patients [21].

BSC is characterized by aggressive features of BCC, which increases the risk of metastasis [22]. The BCC Outcomes with LDE225 Treatment (BOLT) trial indicated a response rate of 59.5% for patients with aggressive features, including BSC, and 55.2% for those with nonaggressive features among BCC subtypes. Despite this, the aggressive and nonaggressive features of BCC patients also have benefit from HHI [23]. This can be explained through the role of the Hh pathway, which involves the pathogenesis of BSC. Additionally, Chiang et al. reported genetic alteration in the Hh pathway, *PTCH1*, *SMO* and *MYCN* mutation from BSC and BCC patients [24]. However, the genetic alteration, including *PTCH1*, *SMO* and *MYCN* mutations, was also reported in patients with BSC who were resistant to HHIs [2].

Immunotherapy was also proposed as a treatment option for BCC patients who did not respond to HHIs. Furthermore, cemiplimab is an anti-programmed cell death-1 (anti-PD-1) treatment that targets the PD-1 receptor using an immunoglobulin G4 monoclonal antibody. Lewis et al. demonstrated the efficacy and safety of cemiplimab in patients resistant to HHIs, with an objective response rate of 22% (95% CI: 12–36%) [25]. In addition, De Giorgi et al. documented a patient with locally advanced BCC who exhibited resistance to HHIs, with a reduction and improvement of lesions observed following six months of treatment with cemiplimab [26]. Recent evidence also investigated the combination of cemiplimab with sonidegib in advanced BCC patients; thus, results in this combination need to be explored in further trials [27]. Conversely, continuing of HHIs for patients who are resistant to HHIs is also recommended beyond immunotherapy, such as anti-PD1 therapy [28]. Thus, further prospective cohort studies should investigate the role of genetic alteration in patients who are resistant to HHIs for improving therapeutic results.

The strength of this review lies in its comprehensive analysis of the prevalence and associated gene mutations among BCC patients resistant to Hh pathway inhibitors. This analysis aids in understanding the benefits and limitations of targeted therapy for these patients.

However, the study has limitations, including a small participant pool and missing baseline data in some included studies. These limitations impact the robustness of the results. Nonetheless, the data can inform additional treatment strategies to overcome resistance mechanisms, leading to better therapeutic outcomes.

## Conclusions

Our comprehensive review identified *PTCH1*, *SMO* and *TP53* as the most prevalent genetic loci associated with resistance to Hh pathway inhibitors.

## Supplementary Material

supplementary.docx

supplementary data 2_search strategy.docx

## Data Availability

The data that support the findings of this study are available from the corresponding author, CL, upon reasonable request.
