# Supplementary material for: Prevalence of genetic alterations in basal cell carcinoma patients resistant to Hedgehog pathway inhibitors: a systematic review
Source: Ann Med. 2025 Jun 16;57(1):2516701. doi: 10.1080/07853890.2025.2516701 (PMC12172076; doi:10.1080/07853890.2025.2516701)
Supplement: supplementary data 2_search strategy.docx [file IANN_A_2516701_SM6948.docx]

**Supplementary Data 2: search strategies**

**Embase**

1. 'vismodegib'/exp OR 'vismodegib'

2. 'sonidegib'/exp OR 'sonidegib'

3. 'hedgehog'/exp OR 'hedgehog'

4. 'basal cell carcinoma'/exp OR 'basal cell carcinoma'

5. bcc

6. hhis

7. shhis

8. 'hedgehog inhibitor'/exp OR 'hedgehog inhibitor'

9. ('sonic'/exp OR sonic) AND ('hedgehog'/exp OR hedgehog) AND ('inhibitor'/exp OR inhibitor)

10. smoothened AND ('inhibitors'/exp OR inhibitors)

11. smoi

12. #1 OR #2 OR #3 OR #6 OR #7 OR #8 OR #9 OR #10 OR #11

13. 'surgery'/exp OR 'surgery'

14. 'radiotherapy'/exp OR 'radiotherapy'

15. #4 OR #5

16. #13 OR #14

17. #12 AND #15 AND #16

**Scopus**

( ( TITLE-ABS-KEY ( sonidegib ) ) OR ( TITLE-ABS-KEY ( odomzo ) ) OR ( TITLE-ABS-KEY ( erismodegib ) ) OR ( TITLE-ABS-KEY ( lde225 ) ) OR ( TITLE-ABS-KEY ( vismodegib ) ) OR ( TITLE-ABS-KEY ( gdc-0449 ) ) OR ( TITLE-ABS-KEY ( erivedge ) ) OR ( TITLE-ABS-KEY ( hedgehog AND inhibitor ) ) OR ( TITLE-ABS-KEY ( sonic AND hedgehog AND inhibitor ) ) OR ( TITLE-ABS-KEY ( hhi ) ) OR ( TITLE-ABS-KEY ( shhi ) ) OR ( TITLE-ABS-KEY ( smoothened AND inhibitors ) ) OR ( TITLE-ABS-KEY ( smoi ) ) ) AND ( ( TITLE-ABS-KEY ( bcc ) ) OR ( TITLE-ABS-KEY ( basal AND cell AND carcinoma ) ) )

**Pubmed**

1. ((((((Sonidegib) OR (Odomzo)) OR (Erismodegib)) OR (LDE225)) OR (NVP-LDE225)) OR (((Vismodegib) OR (GDC-0449)) OR (Erivedge))) OR (Smoothened inhibitors) OR (SMOi)

2. (basal cell carcinoma) AND (basal cell carcinoma[MeSH Terms])

3. BCC

4. ((basal cell carcinoma) AND (basal cell carcinoma[MeSH Terms])) OR (BCC)

5. (surgery) AND (surgery[MeSH Terms])

6. (radiotherapy) AND (radiotherapy[MeSH Terms])

7. ((surgery) AND (surgery[MeSH Terms])) OR ((radiotherapy) AND (radiotherapy[MeSH Terms]))

8. sonic hedgehog inhibitor

9. hedgehog inhibitor

10. Smoothened inhibitors

10. SHHi

11. HHi

12. SMOi

13. ((((((((((Sonidegib) OR (Odomzo)) OR (Erismodegib)) OR (LDE225)) OR (NVP-LDE225)) OR (((Vismodegib) OR (GDC-0449)) OR (Erivedge)))) OR (sonic hedgehog inhibitor)) OR (hedgehog inhibitor)) OR (SHHi)) OR (HHi) OR (Smoothened inhibitors) OR (SMOi)

14. ((((((((((((Sonidegib) OR (Odomzo)) OR (Erismodegib)) OR (LDE225)) OR (NVP-LDE225)) OR (((Vismodegib) OR (GDC-0449)) OR (Erivedge)))) OR (sonic hedgehog inhibitor)) OR (hedgehog inhibitor)) OR (SHHi)) OR (HHi) OR (Smoothened inhibitors) OR (SMOi)) AND (((surgery) AND (surgery[MeSH Terms])) OR ((radiotherapy) AND (radiotherapy[MeSH Terms])))) AND (((basal cell carcinoma) AND (basal cell carcinoma[MeSH Terms])) OR (BCC))

15. ((((((((((((Sonidegib) OR (Odomzo)) OR (Erismodegib)) OR (LDE225)) OR (NVP-LDE225)) OR (((Vismodegib) OR (GDC-0449)) OR (Erivedge)))) OR (sonic hedgehog inhibitor)) OR (hedgehog inhibitor)) OR (SHHi)) OR (HHi) OR (Smoothened inhibitors) OR (SMOi))) AND (((basal cell carcinoma) AND (basal cell carcinoma[MeSH Terms])) OR (BCC))

**CENTRAL**

#1 ("Sonidegib"):ti,ab,kw

#2 ("Odomzo"):ti,ab,kw

#3 ("Erismodegib"):ti,ab,kw

#4 ("LDE225"):ti,ab,kw

#5 ("NVP-LDE225"):ti,ab,kw

#6 ("Vismodegib"):ti,ab,kw

#7 ("GDC-0449"):ti,ab,kw

#8 ("Erivedge"):ti,ab,kw

#9 ("Smoothened inhibitors"):ti,ab,kw

#10 SMOi

#11 ("sonic hedgehog inhibitor"):ti,ab,kw

#12 ("hedgehog inhibitor"):ti,ab,kw

#13 SHHi

#14 ("HHi"):ti,ab,kw

#15 ("basal cell carcinoma"):ti,ab,kw

#16 MeSH descriptor: [Carcinoma, Basal Cell] explode all trees

#17 ("BCC"):ti,ab,kw

#18 {OR #1-#14}

#19 {OR #15-#17}

#20 #18 AND #19
